# Do ABC transporters regulate plasma membrane organization?

**DOI:** 10.1186/s11658-020-00224-x

**Published:** 2020-07-06

**Authors:** Ambroise Wu, Karolina Wojtowicz, Stephane Savary, Yannick Hamon, Tomasz Trombik

**Affiliations:** 1 Faculty of Biotechnology, University of Wroclaw, Wroclaw, Poland; 2 Lab. Bio-PeroxIL EA7270, University of Bourgogne Franche-Comté, Dijon, France; 3 Aix Marseille University, CNRS, INSERM, CIML, Centre d’Immunologie de Marseille-Luminy, Marseille, France

**Keywords:** ABC transporter, Plasma membrane, Cholesterol, Phospholipids, Rafts, Membrane (nano)domains

## Abstract

The plasma membrane (PM) spatiotemporal organization is one of the major factors controlling cell signaling and whole-cell homeostasis. The PM lipids, including cholesterol, determine the physicochemical properties of the membrane bilayer and thus play a crucial role in all membrane-dependent cellular processes. It is known that lipid content and distribution in the PM are not random, and their transversal and lateral organization is highly controlled. Mainly sphingolipid- and cholesterol-rich lipid nanodomains, historically referred to as rafts, are extremely dynamic “hot spots” of the PM controlling the function of many cell surface proteins and receptors. In the first part of this review, we will focus on the recent advances of PM investigation and the current PM concept. In the second part, we will discuss the importance of several classes of ABC transporters whose substrates are lipids for the PM organization and dynamics. Finally, we will briefly present the significance of lipid ABC transporters for immune responses.

**This article was specially invited by the editors and represents work by leading researchers**.

## Introduction

Lipid arrangements determine the physicochemical nature of cell membranes, choreographing signaling cascades and cell metabolism. Deciphering the mechanisms by which cells control the lateral and transversal membrane organization is of general interest considering that auto-immune, atherogenic, or proliferative pathologies might originate from a dysfunction of the lipid equilibrium in the plasma membrane. This field of research is, however, neglected due to its extreme experimental difficulty when considering living cells under physiological conditions. There are indeed several layers of complexity: (a) diversity-related lipid composition (cholesterol, phospholipids, sphingolipids...), (b) their lateral assembly as areas of variable size and composition, (c) the existence of transient interactive proteins and (d) the asymmetry of the two hemi-membranes. Membrane lipid transporters among the members of the ATP Binding Cassette (ABC) transporter and the P-type ATPase families have been hypothesized to be pivotal in the maintenance of membrane lipid composition homeostasis [1]. Although structurally distinct, these transporters share common features such as their polytopic nature, their high molecular weight, and their cytosolic ATPase activity, fueling their transport activity of lipid compounds (for a majority of them). However, establishing the exact nature of their allocrites is still puzzling, since redundant, indirect, or compensatory activities may obscure experimental readouts as demonstrated for multidrug resistance-associated ABC transporters [2]. This review aims to update on recent advances in the knowledge of membrane organization in living cells and how lipid ABC transporters might be implicated in its homeostasis including their direct (associated with the PM) and indirect (function in lipid metabolism that contributes to changes in the composition of membrane lipids) effects on the plasma membrane.

## PM organization: from historical perspectives to recent advances

Over the last 40 years, constant progress has been made to refine the original model of plasma membrane organization, described as the fluid mosaic membrane model by Singer and Nicholson, in 1972 [3]. The plasma membrane was defined as a two-dimensional lipid bilayer (composed of amphiphilic phospholipids) where globular integral proteins were incorporated, which was conceived to unify sparse experimental evidence, contradicting the Davidson-Danielli tri-layer (protein-lipid-protein) model [4]. The fluid-mosaic membrane model incorporated at least two major concepts, which have not been disproved since then, namely the fluidity and dynamics of the membrane components and their mosaic nature, partly based on heterokaryon experiments of human and murine cells [5], whose components diffuse laterally and mix rapidly at physiological temperature. As early as 1976, the fluid mosaic model was enriched by its authors, taking into consideration that the cytoskeleton and extracellular matrix could influence the diffusion of membrane molecules and that ordered, or solid lipid phases could exist [6].

Thus the fluid mosaic model from 1972 was often caricatured as simplistic even though its authors had before everyone else introduced fundamental notions such as the formation of specialized domains of the membrane, allowing the reversible association of proteins or lipids, the sequestration or exclusion of membrane components, and translocation directed by the cytoskeleton [7]. Of course, the biochemical nature of these areas had not been established yet, nor their functional importance. However, at that time, the authors suspected the existence of non-covalent forces between membrane components and the formation of paracrystalline structures, particularly at the tight junction level. Precisely in polarized cells where these junctions separate apical and basolateral membranes, new aspects of the structure of cell membranes have been discovered, leading to the concept of the lipid raft described in the mid-1990s [8, 9]. It had been shown that there was differential lipid trafficking in these cells, where sphingolipids (glycosphingolipids and sphingomyelin) were preferentially apical, while glycerolipids such as phosphatidylcholines were basolateral. The existence of an intracellular sorting center where these apical microdomains composed of packed sphingolipids would be constituted led the authors to postulate the existence of moving platforms created by packing mainly of sphingolipids and cholesterol on which proteins could be selectively included or excluded.

Additionally, it transpires from the studies of artificial biomembranes that lipids can self-associate, conferring a coherent lateral structure. In model membranes, there was observed a phase separation depending on cholesterol added into a mixture with phosphatidylcholine [10]. This leads to lateral segregation of lipid phases, where the polycyclic steroid core of the cholesterol favors its interaction with extended saturated acyl chains of phospholipids, increasing the membrane rigidity locally while excluding phospholipids with bulky unsaturated acyl chains (review in K. Simons et al., [11]). Sphingolipids that harbor longer and more saturated hydrocarbon chains also interact preferentially with cholesterol. Thus in planar lipid monolayers two coexisting lipid phases are created: a thicker lipid ordered phase (Lo), and a thinner lipid disordered phase (Ld). This was extensively studied by atomic force microscopy and fluorescence microscopy (Lin, W. C et al., [12] as an example). Complexifying lipid composition into ternary mixture broadens our knowledge of membrane lipid segregation (reviewed in Marsh, D [13].) while facing the challenge of recreating in vitro the vast diversity of a native plasma membrane, composed of thousands of different lipid species (considering the variability in the length/saturation of acyl chains) and hundreds of various membrane proteins, not to mention the presence of the subcortical actin cytoskeleton.

At that time, the main experimental arguments on the cell plasma membrane rafts were based on the insolubility of glycolipid-enriched complexes to non-ionic detergents such as Triton X-100⁠ at 4 °C, and their flotation property in density gradients [14–16]. How then, to be sure that this method does not itself create these “domains”? Intuitively, apart from the presence of detergents, the temperature is expected to influence the biophysical properties of lipids, and the solvability of proteins, which would divert these “detergent-resistant membranes” (DRM) from the living matter [17]. In retrospect, while the concept provided new experimental directions for the level of organization of the plasma membrane, it was based on potentially questionable analytical methods. In this line, numerous studies have shown that the DRM insolubility may be due not only to its lipid composition but also to the anchoring of proteins to the cytoskeleton [18, 19]. One of the common criticisms expressed regarding the purification temperature could be addressed when other less stringent detergents (e.g. Brij98) made it possible to purify DRMs at physiological temperature [20–23], although showing some notable differences between the methods and the choice of the detergent [24]. Another method emerged, consisting of aggregating these raft lipids with cholera toxin, which binds specifically to GM1 gangliosides, preferentially partitioning in DRMs, and considered as markers of the lipid raft [25]. Stable micrometric platforms were observed in confocal microscopy [25]. However, it was not possible to know whether these aggregated domains were a universal signature of lipid rafts, or whether they were a subspecies of domains, assuming that they existed in physiological conditions without aggregation. As a matter of fact, to know whether these domains existed as stable or dynamic lipid entities and whether they condensed in response to an extracellular signal has always proved to be a challenge. Analysis of the onset of the adaptive immune response in different cells such as T and B lymphocytes has shown in in vitro stimulation systems that signaling molecules with membrane patches were aggregated by cholera toxin [25, 26], and co-purified in Triton X-100 insoluble fractions, in an activating condition with soluble antibodies [27]⁠. Once again, experimental limitations at the time did not allow the existence of these lipid rafts to be established unambiguously. The Laurdan lipid probe was also detected in condensed areas of the Jurkat leukemic T cell membrane at the activation sites by beads coated with stimulating antibodies [28, 29]. This probe possesses the property of being inserted parallel to the phospholipids, without partitioning into ordered rather than disordered liquid phases, but whose spectral properties change if it is inserted into condensed regions of a bilayer. The extrusion of cholesterol by methyl-beta-cyclodextrin inhibited this condensation [30]. But other experimental data using this compound have shown that extracting membrane cholesterol may have an inhibitory effect on signaling phenomena [31], have a negative effect on cell survival [32], or target free cholesterol in non-lipid raft fractions [32]. Methyl-beta-cyclodextrin should be carefully considered as a disturbing agent of the membrane, not devoid of side effects. Taken together, these data seemed to show that rafts could constitute signaling platforms whose aggregation impacts on signal transduction in the immune cells studied (T lymphocytes). However, many contradictory data populate the scientific literature concerning lipid rafts, leading to the questioning of their very existence. One of the recurring questions is whether these lipid domains are only the experimental consequence of observation methods based on their direct or indirect aggregation, and, by extension, whether they exist in the physiological state.

The introduction of advanced biophysical techniques (single-particle tracking, fluorescence correlation spectroscopy (FCS), fluorescence recovery after photobleaching) has provided new arguments for the existence of isolated membrane domains of nanometric size, enriched with cholesterol and sphingolipids, and of a highly dynamic nature [16, 33]. The major advantage of these approaches is that they can be performed on living cells in physiological conditions, minimizing disruptions of cellular systems. It appeared that it was possible to probe the organization of the membrane by measuring the diffusion time as a function of the diameter of the confocal excitation spot [34–36]. This method, called spot variation FCS, has shown, based on experimental measurements and numerical simulations, that FCS diffusion times follow a linear relationship with the illumination surface, but deviate from an affine relationship as in the case of Brownian diffusion [37, 38]. These deviations reflect the nature of the dominant confinement of the molecules observed, either in isolated domains or by a meshwork, mostly referring to the cortical actin cytoskeleton. These results were confirmed at different spatial scales in stimulated emission depletion (STED) FCS and scanning FCS and applied to various biological situations [39–42]⁠⁠. Biophotonic techniques have been the best experimental approach to highlight the existence of dynamic membrane inhomogeneities, together with single-particle tracking (hop diffusion) [43, 44], electron paramagnetic resonance of spin-labeled phospholipids and cholesterol analogues (reviewed in Subczynski, W. K., & Kusumi, A [45].), STED [40, 46] or fluorescence lifetime imaging microscopy (actin asters) [47, 48]. On the one hand, it appears that lipid raft marker molecules, such as glycosylphosphatidylinositol-anchored proteins, are indeed molecules whose diffusion is not purely Brownian [35, 49], ensuring an overall consistency to all these approaches, even if the biochemical nature of their confinement is a matter of debate [50]. What is important to note is that there is not one type, one size, one lifetime of membrane domains, but a multitude of biochemical confinement nature (lipid-lipid, actin domains, lipid-protein, etc.) and that there is not a single type of lipid domain, but each molecule may have its signature type of confined diffusion, based on their interactions, not exclusively with cholesterol and sphingolipids, but also with their local environment. For example, some anionic phospholipids such as phosphatidylserine or polyphosphoinositides (e.g. PIP2 or PIP3) seem to play a role in the specialization of nanodomains [44], in relation with a structural configuration of the proteins with which they interact [51, 52]. This notion of specialization and diversity, reminiscent of the lipid shell concept [53], would ensure the homeostasis of cellular systems restricting access of reaction patterns to potential interlocutors in the absence of specific activation signals [54, 55].

It has long been tempting to envisage ATP binding cassette (ABC) transporters as master players in lipid rafts and, by extension, plasma membrane organizers. These transporters belong to five different subfamilies (from A to G) and have distinct substrate specificities and subcellular localizations [56].

## Cholesterol effluxes in lipid domains may affect membrane lipid organization

**Fig. 1:**
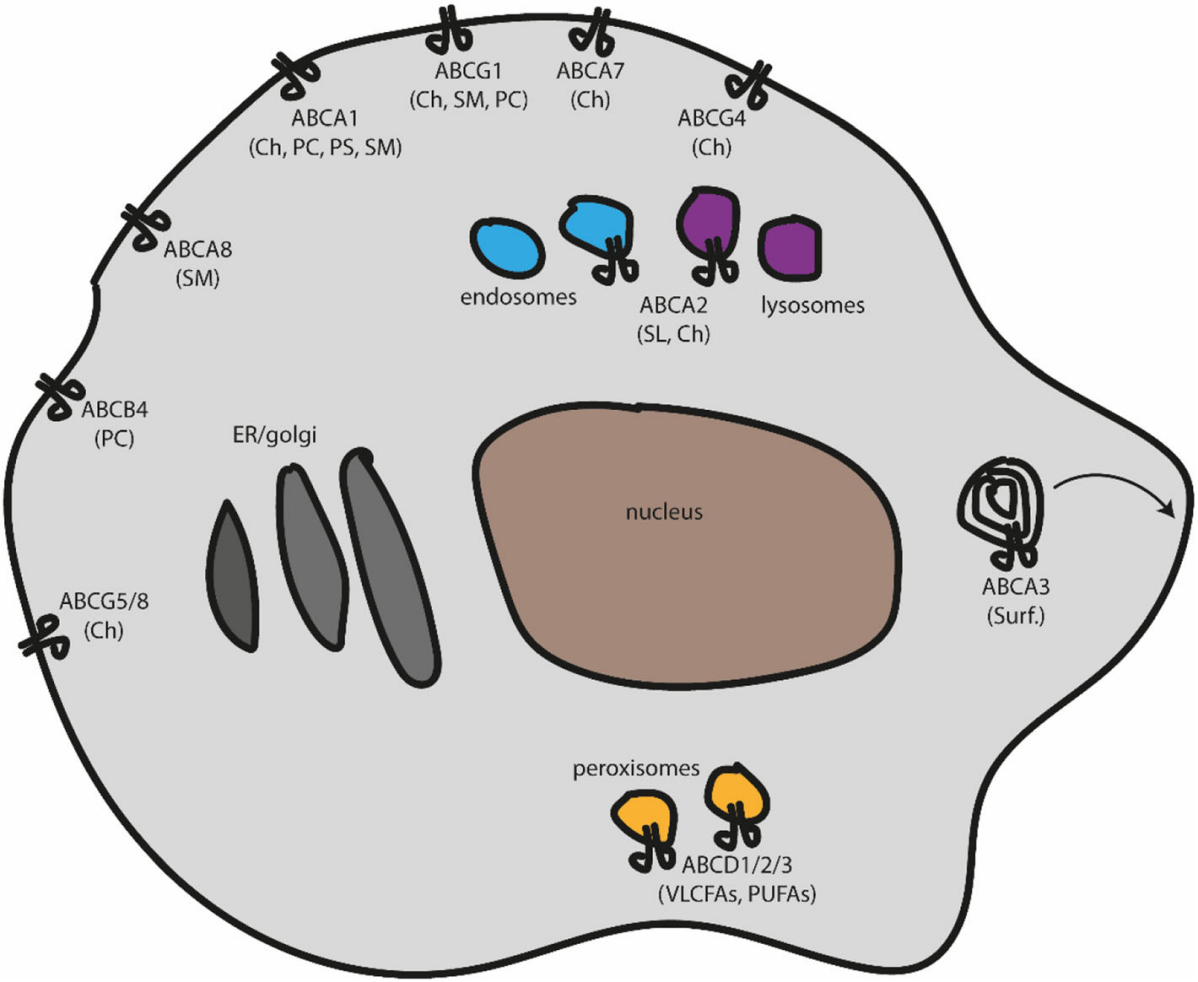
Schematic representation of the cell with localization of lipid-related ABC transporters together whit their substrates (in parenthesis). Ch – cholesterol; PC – phosphatidylcholine; PS – phosphatidylserine; SM – sphingomyelin; SL – sphingolipids; Surf. – surfactant; VLCFAs – very-long-chain fatty acids; PUFAs – polyunsaturated fatty acids

On the grounds of their transport function, acting as lipid floppases or membrane lipid exporters, it has been repeatedly suggested that ABC transporters could be such central regulators of the plasma membrane. Few biophysical studies support this view [57]. Most evidence arises from DRM methodologies, consistently showing that ABCA1 is solubilized by Triton X-100 but not ABCG1, whereas apolipoprotein A-I (ApoA-I)-mediated cholesterol effluxes would take place in lipid rafts dependent upon ABCA1 functionality in macrophages [58]. Cholesterol is tightly regulated at the PM, as a result of intracellular trafficking, lateral distribution, and efflux towards plasmatic acceptors. The most widely described phenomenon is indeed cholesterol efflux mediated by ABCA1 and ABCG1 (Fig. 1). ABCA1, the prototype of the ABCA subfamily, is ubiquitously expressed, tightly regulated by the liver X receptor/retinoid X receptor pathway [59], concomitantly with ABCG1. It has a pivotal role in the removal of cholesterol from peripheral tissues to the liver in the reverse cholesterol transport pathway [60]. Mutations in the *ABCA1* gene lead to a genetic disorder named Tangier disease, whose main hallmarks are low level of high-density lipoprotein (HDL) molecules, the accumulation of cholesterol in peripheric tissues, pronounced atherosclerosis and premature coronary artery disease [61, 62]. It has been shown that ABCA1 drives lipid efflux for HDL biogenesis mediated by the lipidation of lipid poor ApoA-I [63], and this process occurs in the PM lipid rafts [64]. It is unclear nonetheless whether ABCA1 and ApoA-I physically interact despite the fact that chemical cross-linking between ApoA-I and ABCA1 has been evidenced [65]. Another alternative model would be that ApoA-I could interact with the PM in dedicated regions created upon the ABCA1 function [63, 66, 67].

ABCG1 is also a central protein for intracellular sterol homeostasis in a variety of cells, including macrophages, neurons, and endothelial cells. It has been demonstrated that the physiological role of ABCG1 includes cholesterol transport from cells to lipid extracellular acceptors, which is regulated by the liver X receptor/retinoid X receptor pathway [68]. In contrast to ABCA1, ABCG1 promotes sterol efflux to various relatively nonspecific acceptors such as HDL, low-density lipoprotein (LDL), and cyclodextrin, but not to ApoA-I [69]. Although several authors have argued that ABCG1 is mainly localized in endosomes, it is now clear that ABCG1 traffics to the plasma membrane, where it increases the cholesterol cell removal [70, 71], apparently tightly in contact with actin cytoskeleton [72].

Due to its high homology with ABCA1 (54% based on amino acid sequence), ABCA7 was suggested to share with ABCA1 the role of a lipid exporter [73]. However, even if it has been shown that in ABCA7 over-expressing HEK 293 cells the transporter bound to a cholesterol acceptor, ApoA-I, it mediated only the efflux of cellular phospholipids but not cholesterol, whereas ABCA1 mediates both [74], resulting in the generation of mostly cholesterol-poor HDL particles [75]. Moreover, in *ABCA7*^−/−^ mice macrophages phospholipid and cholesterol effluxes did not appear to be altered while ABCA1 loss of function prevented these effluxes [76]. This suggests that ABCA7 plays an accessory role in lipid trafficking compared to ABCA1. However, *ABCA1*-deficient mouse fibroblasts presented higher expression of ABCA7 [77], while *ABCA7*-deficient mouse macrophages displayed an increase of ABCA1 expression [78], implying that ABCA1 and ABCA7 could in certain atypical circumstances compensate the loss of one another.

Other members of the ABCG subfamily have been implicated in sterol transport (Fig. 1), mostly exemplified by the ABCG5/8 hemi-transporters, which form a functional heterodimer at the canalicular membrane of hepatocytes and promote biliary excretion of sterols into the gallbladder, being the final bile reservoir [79], and causing sitosterolemia when mutated [80]. ABCG4 is another member of the family of ABCG transporters, which were discovered initially based on the high sequence homology with ABCG1. These proteins are closely related both in amino acid sequence and in specific functions such as cholesterol efflux to HDL but not to ApoA-I. It has been demonstrated that both ABCG1 and ABCG4 can form a heterodimer and cooperate to remove cellular cholesterol efficiently [81]. Unlike ABCG1, ABCG4 is unresponsive to LXR activation, and its expression concerning cell types and tissues is significantly restricted [68]. ABCG4 is highly expressed in the brain, in particular in neurons or astrocytes, and it can play an indirect role in Alzheimer’s disease, which is related to disrupted cholesterol metabolism and amyloid-β-peptide accumulation [82].

It has been proposed that ABCA1 synergizes sequentially with ABCG1/G4 to increase the formation of HDL and optimize cellular cholesterol efflux. In this context, HDL-like particles formed by the activity of ABCA1 act as acceptors of cholesterol exported by either ABCG1 or ABCG4 proteins. Moreover, the nascent lipoprotein particles produced by ABCA1 are capable of cholesterol removal from cell surface domains formed by either ABCG1 or ABCG4 transporters [81]. All of the mentioned proteins seem to be very important for cholesterol and lipid homeostasis; however, they could modulate various physiological interactions occurring within the plasma membrane and disrupt lipid raft structures, modulating the plasma membrane organization [83].

## ABC transporter dependent-phospholipid flopping affects membrane organization

Several mammalian ABC transporters share the property of translocating phospholipids from the inner leaflet to the outer leaflet (floppase activity) with generally poor specificity. This feature probably originates from the very first ABC transporter, P-glycoprotein (ABCB1), conferring to the cells the ability to extrude cytotoxic drugs, mainly hydrophobic, embedded in the plasma membrane. This multidrug resistance property is widely unspecific and encompasses hundreds of different chemical compounds. This broad diversity of extruded compounds is additionally increased if other multidrug resistance genes such as *ABCB4*, *ABCC1*, or *ABCC2* are jointly expressed (in particular in the blood-brain barrier, in the liver or along the gastrointestinal tract). This raises the question to what extent ABC transporters share selectivity as lipid translocators and to what extent transbilayer movements of lipids are directly performed by those transporters in physiological conditions. ABCA1 has been shown to transport phosphatidylcholine (PC), phosphatidylserine (PS) and sphingomyelin (SM) (Fig. 1) from the inner leaflet to the outer leaflet of the plasma membrane [63, 84], which may contribute to local heterogeneity suitable for ApoA-I [85] and cholesterol extraction. Associated with this, in an overexpression system in knockout mice, PS exposure activity was correlated with ABCA1 expression [86]. Formal demonstration of lipid translocation activity in living cells is extremely complicated from a technical point of view. An insight into the ABCA1 mechanism of lipid transport has been described by fluorescence lifetime imaging microscopy experiments providing the first evidence that ABCA1 increases the lipid packing of the outer leaflet, altering cholesterol present in the lipid raft within the plasma membrane [57]. These studies provided strong evidence of the role of ABCA1 in plasma membrane lipid organization. Recently, the ABCA1 cryo-electron microscopy structure has been elucidated, revealing a narrow, elongated tunnel formed by the ABCA1 extracellular domain that could potentially translocate phospholipid through the plasma membrane, thus presenting new insights in ABCA1 mechanisms [87]. However, questions regarding the mechanism of cholesterol translocation still remain unanswered. In our recent study, we demonstrated that ABCA1 facilitates the efflux of membrane cholesterol to amphotericin B (AmB), a polyene antibiotic, leading to the formation of bulk cholesterol-AmB structures out of the cell and thus preventing AmB cytotoxicity [88]. From this observation, we can assume that the ABCA1-mediated cholesterol efflux may operate without any specificity for extracellular cholesterol acceptors. In this line, ABCA1 would only create a specific lipid microenvironment from which cholesterol molecules might be easily extracted by extracellular acceptors.

Besides sterols, ABCG1 mediates the efflux of various choline phospholipids, preferentially SM, compared with PC (Fig. 1). Moreover, Hirayama and collaborators have shown that the cholesterol efflux by ABCG1 is dependent on the cellular SM level, which can regulate the ATPase activity of the transporter [89]. As SM tends to form complexes with sterols in the outer leaflet of the plasma membrane, it has been suggested that ABCG1 can move SM together with cholesterol, destabilizing the lipid bilayer and altering membrane organization [70, 90]. Since both SM and cholesterol are essential components of lipid rafts within the plasma membrane, it has been proposed that the ABCG1 activity may be connected to the presence of the nanodomains, as mentioned above [91], although definitive evidence is still lacking.

Additionally, other ABC transporters (Fig. 1) have been implicated in lipid homeostasis and transport in specific cell populations such as ABCA8, which stimulates sphingomyelin production in oligodendrocytes and would affect myelin stability [92]. ABCB4 basically flops PC from the inner to the outer leaflet of the hepatocytes canalicular membrane in the liver. It makes this phospholipid available for extraction by bile salts into the canalicular lumen, where it protects membranes of cells facing the biliary tree against these bile salts by reducing the detergent activity of the bile salt micelles [93].

## Intracellular ABC transporters affect membrane lipid organization

Besides membrane ABC transporters, it has to be emphasized that several intracellular ABC transporters (Fig. 1) have been shown to affect membrane lipid composition indirectly, and by extension PM lateral organization. For example, ABCA2, highly expressed in the brain, plays a role in sphingolipid homeostasis by modulating the intracellular metabolism of sphingolipids [94, 95]. ABCA2 inactivation in mice leads to an age-related modification of brain lipids, resulting in deficiencies in phosphatidylethanolamine (PE), phosphatidylserine, and sphingomyelin and accumulation of ganglioside GM1. ABCA2 is mainly localized in the late endosome/lysosome compartment and the trans-Golgi network in neurons and oligodendrocytes [96], suggesting that ABCA2 may play a role in intracellular lipid trafficking. ABCA2 has also been found to play a role in cholesterol homeostasis. Indeed, ABCA2 over-expression in CHO cells (CHOA2) led to an increased level of unesterified cholesterol in cytoplasmic and endosome/lysosome vesicles together with reduced LDL-derived cholesterol trafficking to the endoplasmic reticulum for cholesterol esterification. ABCA2 over-expression induces a phenotype similar to cholesterol depletion in cells that sequester unesterified cholesterol into endo-lysosomal compartments to prevent cholesterol trafficking back to the plasma membrane [95], due to an imbalanced ceramide/sphingosine ratio within the intraluminal membrane lipid bilayer. It has been reported that in neuronal Schwann cell lines expressing a high level of ABCA2, ceramide metabolite levels were reduced, whereas sphingosine levels were increased [97]. ABCA2 might, therefore, modulate the ceramide/sphingosine ratio by altering lipid metabolism together with esterification of plasma membrane-derived cholesterol [98].

It should be mentioned that high cholesterol level in the brain leads to release of amyloid β protein, whose extracellular accumulation causes Alzheimer’s disease [99]. Different mutations of ABCA2 have been associated with high risk for Alzheimer’s disease [100, 101], but up to now, the mechanistic role of ABCA2 in Alzheimer’s disease remains unclear.

Another intracellular ABC transporter which affects membrane lipid homeostasis is ABCA3. This transporter is mainly expressed in alveolar type II pneumocytes (AT2) in the lung, intracellularly located in lamellar bodies [102]. These secretory organelles are responsible for the storage of pulmonary surfactant, a mixture of cholesterol, phospholipids, and proteins. ABCA3 has been reported to transport lipids into the lamellar bodies of AT2 cells [103]. Indeed, in vitro and in vivo studies have demonstrated that ABCA3 is involved in regulation of the transport of cholesterol, phosphatidylglycerol, PC, SM, PE, and PS, and that *ABCA3*^*−/−*^ mice have shown a loss of pulmonary surfactant in the alveolar space linked to a diminution of lamellar body formation and a reduction of phospholipids in the lung [104]. These studies suggest that ABCA3 directly regulates the lamellar body’s membrane lipid composition by lipid flip-flop of phosphatidylcholine [105]. More details on ABCA3 will help to investigate neonatal lung diseases further, as its functional loss leads to death shortly after birth because of a fatal deficiency in surfactant [106].

## Peroxisomal ABC transporters and membrane lipid homeostasis

Although peroxisomal ABC transporters have no direct effects on PM organization, they greatly contribute to lipid catabolism, providing substrates for alpha- and beta-oxidation and thus participating in whole lipid metabolism. Within the D subfamily of ABC transporters, three half-transporters called ABCD1, ABCD2, and ABCD3 are expressed at the peroxisomal membrane (Fig. 1), whereas ABCD4, the transporter of cobalamin, is expressed at the lysosomal membrane after translocation from the endoplasmic reticulum [107]. ABCD1 was shown to transport coenzyme A-esters of saturated and monounsaturated very-long-chain fatty acids (VLCFAs, fatty acids with more than 22 carbon atoms), and its defect is linked to X-linked adrenoleukodystrophy (X-ALD), the most frequent peroxisomal disorder [108–110]. ABCD2, the closest homolog of ABCD1, displays a partial functional redundancy with ABCD1 and is also predicted to transport polyunsaturated fatty acids (PUFAs) [111, 112]. ABCD3 was recently associated with congenital bile acid synthesis defect-5 and is thought to transport C27-bile acid intermediates but also dicarboxylic acids and branched-chain fatty acyl-CoAs into the peroxisomal matrix [113]. Of note, beta-oxidation is also part of anabolic reactions leading to the synthesis of PUFAs such as docosahexaenoic acid, one of the most essential fatty acids in nervous tissues.

The impact of peroxisomal ABC transporters, and more extensively of peroxisomal metabolism, on membrane structure and function has largely been overlooked. Peroxisomal defects are associated with alterations in the contents of various lipids of importance for membrane functions: fatty acids, plasmalogens (ether-lipids), cholesterol [114]. The recent understanding that peroxisomes are not only metabolically connected with other cell compartments, but also interact closely with them, underscores the importance of peroxisomes in lipid exchanges and membrane lipid homeostasis [115]. Many peroxisomal disorders are associated with neurodegenerative processes and defects in myelin, a plasma membrane extension produced from oligodendrocytes, which wraps neuron axons [116–118]. Myelin is rich in VLCFAs and plasmalogens, and its lipid composition depends on fatty acid synthesis but is also very dependent on peroxisome metabolism [119, 120]. Concerning X-ALD, it has long been established that ABCD1 deficiency leads to the accumulation of VLCFAs, cholesteryl esters and also membrane lipids (PC, SM, gangliosides, myelin), as observed in erythrocytes [121, 122], fibroblasts [123], myelin [124] or brain tissues [125, 126] of X-ALD patients. Diagnosis of X-ALD, which was initially based on plasma levels of C26:0, proved to be more accurate when using C26:0-lysoPC quantification from a blood spot [127]. A recent lipidomic study in human fibroblasts with peroxisomal defects, including X-ALD, detailed the accumulation of VLCFAs in phospholipid species (PC and lysoPC, PE, and plasmalogens) [128]. Moreover, brain phospholipids and lysophospholipids from *ABCD1*-deficient mice demonstrated saturated and monounsaturated VLCFA accumulation, mostly at the *sn-1* position of PC and PE [129]. Interestingly, some increased levels of PUFAs were also observed in PC and PE, while PC with an odd-numbered fatty acyl chain and some species of phosphatidylserine, phosphatidylinositides, and phosphatidylglycerol were less present in *ABCD1*-deficient brain [129]. In X-ALD, besides impaired beta-oxidation and increased elongation, a very slow dissociation rate of VLCFAs from a phospholipid bilayer likely contributes to the VLCFA accumulation in membranes [130]. Concerning ABCD2, the modulation of its expression in stable transfectant hepatoma cell models was also shown to trigger modifications of VLCFA levels in phospholipids [111]. Altogether, these data indicate that peroxisomal ABC transporters, at least ABCD1 and ABCD2, contribute to the content of saturated, monounsaturated, and polyunsaturated VLCFAs in membranes, especially in complex lipids such as sphingolipids and plasmalogens. This accumulation likely changes membrane properties and contributes to the pathogenesis of peroxisomal leukodystrophies [131–133]. Of note, in addition to VLCFA accumulation, a ABCD1 defect was found to contribute to accumulation of cholesterol, another modulator of membrane properties [134–136]. Peroxisomes were indeed shown to interact with ER, lysosomes, mitochondria, and lipid droplets and participate in cholesterol trafficking [137, 138].

## ABC transporter-dependent lipid rearrangement and immunity

**Fig. 2:**
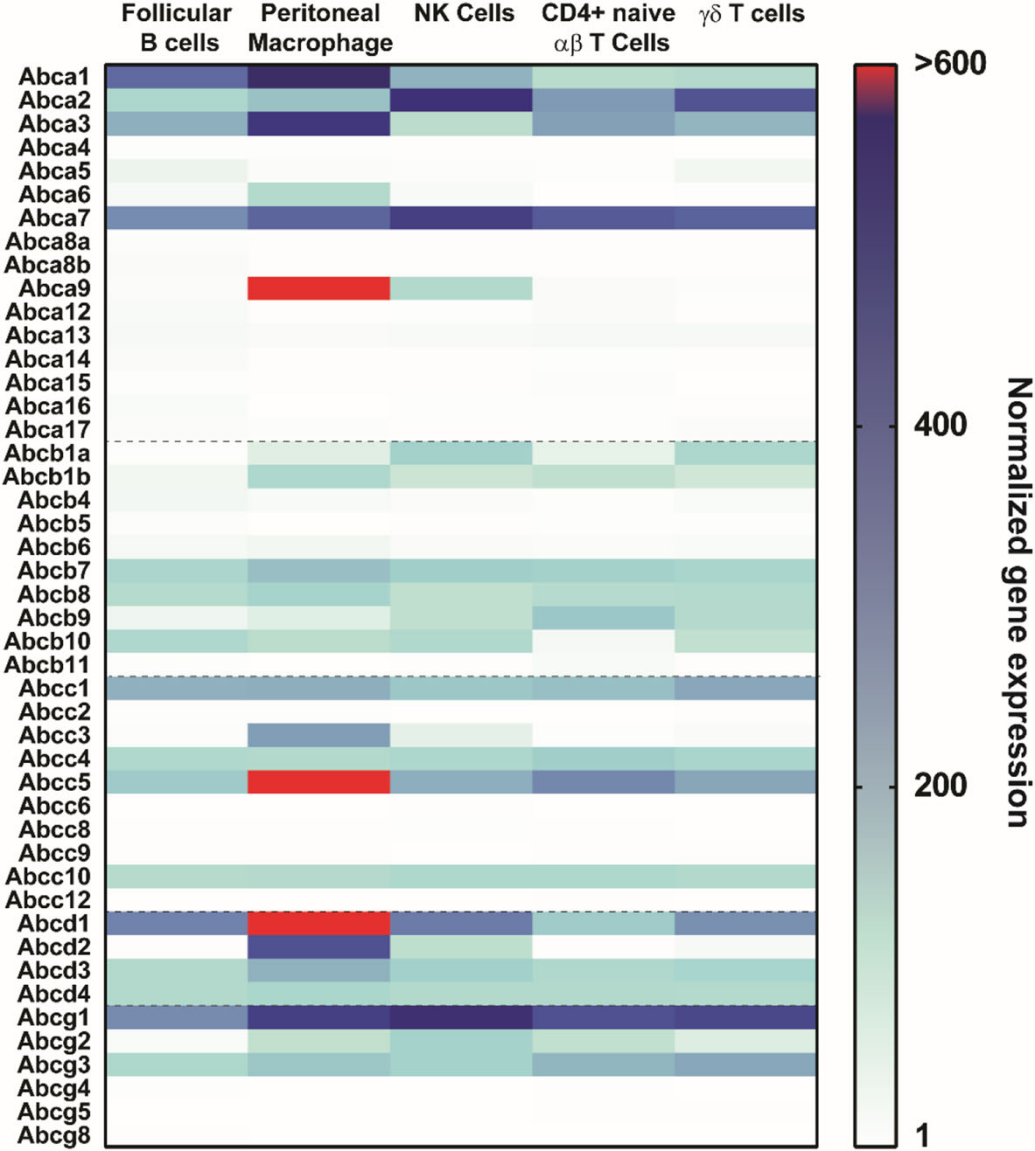
Heat map display of murine ABC transporter normalized expression level in five highly purified immunocyte populations based upon ImmGen Deep RNA-seq data [151] (GEO accession: GSE122597), namely NK Cells, Follicular B, Naive CD4+, αβT, γδT cells, and peritoneal macrophages

It remains to be established whether ABC transporters are master planners of dynamic membrane architecture or limited to individual metabolic functions such as cholesterol transport that indirectly affect other signaling pathways. This is extremely important from an immunological point of view, for example, where immune responses have to be tightly controlled, and this control often occurs by the PM-mediated rearrangement of the cell surface receptors between raft and non-raft structures which may control their activity [139, 140]. While lipid membrane organization has been studied in the context of signal transduction in immune cells, in particular in TCR signaling [21, 25, 141, 142, 162], the role of ABC transporters in this process has been poorly addressed. Inactivation of LXRβ as a major nuclear receptor controlling cholesterol homeostasis shows a major increase in the proliferation of CD4 and CD8 T lymphocytes, which was associated with abolished regulation of ABCA1 and ABCG1 expression upon CD3-crosslinking [143]. It was further confirmed that ABCG1 negatively controls thymocyte and peripheral T lymphocyte proliferation, correlated with an increase in cholesterol cell content [142]. More recently, ABCA1 and ABCG1 have been shown to play a role in interleukin 4 (IL-4) mediated macrophage activation in tumor-associated macrophages [144]. In general, ABCA1/G1 negatively regulates IL-23 secretion from macrophages and dendritic cells, controlling hematopoietic stem and multipotential progenitor cell proliferation [145]. Inactivation of the expression of ABCG1 negatively regulates the secretion of IL-4 in invariant natural killer T lymphocyte cells but positively regulates interferon-gamma production [146], while ABCA1 interferes with IL-4 and interferon-gamma-dependent signal transduction in macrophages [147]. Selective inactivation of ABCG1 in regulatory T cells led to downregulation of the mTOR pathway, correlated with intracellular cholesterol accumulation [148]. The invalidation of ABCA1 and ABCG1 in dendritic cells has been proven as instrumental in cholesterol accumulation in those cells, promoting NLRP3 inflammasome activation and autoimmune pathology through the enhanced secretion of IL-6, IL-12, and IL-23 from *ABCA1/ABCG1*-double knock-out dendritic cells [149]. In this view, ABCA1 and ABCG1 seem to be central regulators of innate and adaptive immune responses, although the precise molecular mechanism is still not fully unraveled. It has also been reported that ABCA7 regulates NKT cell function by controlling the CD1d localization into the lipid rafts and proper cytokine release in response to antigen stimulation [150]. Interestingly though, the careful monitoring of the expression of all members of the mouse ABC transporters in selected immune cell populations (Fig. 2, based on the ImmGen consortium RNAseq data [151]) demonstrates that numerous ABC transporters show a medium to high expression level while having never been experimentally questioned. *ABCG1*, but also *ABCA7*, are highly expressed in different lymphoid and myeloid cell types, while *ABCC5* and *ABCD1* are especially highly expressed in peritoneal macrophages.

## Conclusions and perspectives

In conclusion, much remains still to be elucidated on the role of ABC transporters in immunity, especially in association with cell cholesterol homeostasis and membrane organization integrity. Recent advances in the design and application of molecular probes for cholesterol may help to determine more precisely the relationship between ABC transporters and membrane organization in the future [152, 153].

In a more perspective view, an interesting future research direction may concern the relation between PM organization and tumor development. It has already been demonstrated that the PM content and organization are significant in terms of cancer development and metastasis [154]. Expression and function of several integral PM and PM-associated proteins, which are not randomly distributed over the PM but instead confined to cholesterol- and sphingolipid-rich nanodomains, are altered in cancer cells [155–159]. Moreover, it has been shown that cholesterol content in tumor cells is higher than in healthy cells and is accumulated specifically in lipid raft nanodomains [160]. Finally, tumor-derived exosomes, which are now believed to play a crucial role in pre-metastatic cancer niche formation, originate from lipid raft structures [161]. We can, therefore, assume a possible role of different ABC lipid transporters in these processes. For example, a direct link between ABCA1 and ABCG1 expression and carcinogenesis was recently demonstrated in an ovarian cancer model, where ABCA1/G1-mediated cholesterol efflux from the PM of macrophages promotes tumor progression [144].

Taking all this evidence together, there is no doubt about the importance of the lipid ABC transporters for various cellular processes, and future progress in their investigation is needed to broaden our understanding of single-cell and organism physiology in health and disease.

## Data Availability

Not applicable.

## Abbreviations

ABC: ATP-binding cassette transporter
AmB: Amphotericin B
ApoA-I: Apolipoprotein AI
DRM: Detergent resistant membranes
FCS: Fluorescence correlation spectroscopy
HDL: High-density lipoprotein
IL: Interleukin
LDL: Low-density lipoprotein
LXR: Liver X receptor
PC: Phosphatidylcholine
PE: Phosphatidylethanolamine
PM: Plasma membrane
PS: Phosphatidylserine
PUFAs: Polyunsaturated fatty acids
RXR: Retinoid X receptor
SM: Sphingomyelin
STED: Stimulated emission depletion
VLCFAs: Very long-chain fatty acids
X-ALD: X Linked adrenoleukodystrophy
